# 845 Optimizing the Complex Cell Dialogue During Tissue Repair Processes by Using Rapid Vascularized Collagen/elastin Matrix

**DOI:** 10.1093/jbcr/iraf019.376

**Published:** 2025-04-01

**Authors:** Markus Oehlbauer

**Affiliations:** Burn Center BG Trauma Center Murnau

## Abstract

**Introduction:**

Fibroblasts are key players for maintaining skin homeostasis and for orchestrating physiological tissue repair.

Keratinocytes play an important role in cutaneous cell-cell communication for wound healing outcome. Rapid vascularized tissue matrix, consisting of native collagen (collagen type I, III and V) supplemented by an elastin hydolyzate is used since more than 20 years especially as dermal template. We here report our - after 20-years’experience - optimized setting using this tissue matrix for defect coverage especially in complex wounds and its impact in keraticocyte-fibroblast crosstalk.

**Methods:**

Different settings of wound bed preparation with and without using native collagen-elastin tissue matrix were compared intra-individual and to patients treated by these different settings in our level I trauma center since 2003. Outcome quality of the scar tissue was assessed using electron mikoscope.

**Results:**

Healthy human fibroblasts showed best cell proliferation and collagen synthesis when precise debridement for wound bed preparartion including complete removal of granulation tissue was performed just before application of native collagen/elastin tissue matrix. Long follow up of these native collagen-elastin matrix procedures in defect coverage showed much faster maturation of the scar and excellent functional outcome without occurence of unstable scaring or requirement of scar revision.

**Conclusions:**

The intimate dialogue between the fibroblasts and the native collagen-elastin tissue matrix represents a fascinating domain that must be understood in order to characterize the therapeutic targets especially to prevent pathological developments of myofibroblasts but also to interfere with keraticocytes to induce cutaneous cell-cell communication.

**Applicability of Research to Practice:**

In clinical settings precise debridement for wound bed preparartion before application of native collagen-elastin tissue matrix has shown to be the crucial key point to maximize quality and function of reconstructed tissue.

**Funding for the Study:**

N/A

